# A Prospective Cohort Study of Cutaneous Leishmaniasis Risk and Opium Addiction in South Eastern Iran

**DOI:** 10.1371/journal.pone.0089043

**Published:** 2014-02-20

**Authors:** Mohammad Reza Aflatoonian, Iraj Sharifi, Maryam Hakimi Parizi, Ali Reza Fekri, Behnaz Aflatoonian, Maryam Sharifi, Ahmad Khosravi, Ali Khamesipour, Hamid Sharifi

**Affiliations:** 1 Neuroscience Research Center, Kerman University of Medical Sciences, Kerman, Iran; 2 Leishmaniasis Research Center, Kerman University of Medical Sciences, Kerman, Iran; 3 Research Center of Tropical and Infectious Diseases, Kerman University of Medical Sciences, Kerman, Iran; 4 Kerman Oral and Dental Diseases Research Center, Kerman University of Medical Sciences, Kerman, Iran; 5 Center for Research and Training in Skin Diseases and Leprosy, Tehran University of Medical Sciences, Tehran, Iran; 6 Regional Knowledge Hub, and WHO Collaborating Centre for HIV Surveillance, Institute for Futures Studies in Health, Kerman University of Medical Sciences, Kerman, Iran; 7 Department of Food Hygiene and Public Health, Faculty of Veterinary Medicine, Shahid Bahonar University of Kerman, Iran; Wake Forest University Health Sciences, United States of America

## Abstract

Opium addiction and cutaneous leishmaniasis (CL) are endemic in different parts of Iran, particularly in Bam, where a massive earthquake occurred. This study was designed to compare the incidence rate and severity of CL cases among opium addicted and non-addicted individuals in south-eastern Iran. This study was carried out as a prospective cohort by active house-to-house visits of 1,481 habitants in Bam. CL cases were confirmed by smear and identification of *Leishmania* species was performed using nested-PCR. The data was analyzed by χ^2^ and t-tests, using SPSS software and also Kaplan-Meier survival curve and long-rank test in Stata 11.2 and *P*<0.05 was considered as significant. A total of 904 individuals consisting of 226 opium addicted and 678 non-addicted individuals were followed-up for a period of seven years. The two cohorts were similar in terms of age, sex and place of residency. A similar pattern of incidence was observed among the two cohort groups. In contrast, the severity of CL in terms of the number, duration and the size of the lesions in opium addicted individuals was significantly (*P*<0.001) higher than non-opium addicted individuals. In conclusion, the present findings indicate that there is no relationship between the incidence of CL and opium addiction.

## Introduction

Opium abuse is a serious public health threat in Iran and in neighbouring countries. Opiates are the most commonly used drugs, notably in Asia and Europe, while heroin is the most widely consumed opiate, worldwide [1]. Two thirds of opium which is not converted into heroin is consumed in just five countries [2]: Iran (42%), Afghanistan (7%), Pakistan (7%), India (6%) and the Russian Federation (5%). According to the United Nation World Drug Report (UNWDR), the highest proportion of opium addiction in the world is reported from Iran; it is estimated that 2.8% of the population over age 15 years old are addicted, while the world consumption rate is about 0.5% [3]. As a pain killer opiates have been used in Iran for centuries, although use and trade are officially illegal with harsh penalties, opiate trafficking and consumption are major health problems in the country [4]. The current situation forced the government to implement a number of actions including support and treatment for addicts and rehabilitation centers to initiate prophylactic measures [5].

Leishmaniasis is reported in 98 countries and territories, with prevalence of 12 million people and annual incidence of about 2 million [6]. Cutaneous leishmaniasis (CL) makes up 75% of the total cases in which 90% of the patients are from Afghanistan, Algeria, Iran, Iraq, Saudi Arabia, Syria, Brazil and Peru [7]. Leishmaniasis is among the emerging diseases which has recently had an enormous impact on public health aspects and well-being [6], [8]. CL exists in two epidemiological forms: zoonotic CL (ZCL), due to *Leishmania major* which presents in rural areas where the gerbil is the main reservoir host with *Phlebotomus papatasi* as a vector. Anthroponotic CL (ACL) is caused by *L. tropica* in large and medium size cities, where humans are the main reservoir host and *P. sergenti*, the main vector. The city of Bam is a well-known focus for ACL where it has been endemic since ancient times [9]. Recent surveillance shows that opium addiction and CL incidence have significantly increased in different provinces of Iran during the last decade [3], [10]. The increase in CL incidence and expansion of the disease to new regions are attributed to new settlements, urbanization, agricultural development, migration, improvement of reporting systems and ecological changes such as earthquakes [11], [12], [13]. Among the opioids, opium is the major drug of abuse (over 85%), mainly among males in Bam, and the ratio of males to females is 13.5 to 1.

The Bam district is located in the south-eastern province of Kerman, 1000 m above sea level and covers an area of 168,000 km^2^ with a population of 250,000. A major 6.6 Richter scale earthquake struck Bam and the neighbouring areas on 26^th^ December, 2003. The earthquake was particularly destructive, resulting in approximately 30,000 deaths, 60,000 homeless and injuring an additional 30,000 and over 90% of the medical and health infrastructures were destroyed [14].

Psychologically, the earthquake had a profound impact on its victims and not just in the immediate aftermath [15]. Opium is widely used as a pain killer [16] and Bam is located on the traffic trade route from Afghanistan and Pakistan and as such opium is easily available.

There are studies on prevalence of opium abuse in Iran [17]–[19], but to our knowledge there is no study to evaluate the effect of opium on CL, nationally or abroad. The objective of the present study was to assess the relationship between CL incidence and the severity of the disease among opium addicted and non -opium addicted individuals in the Bam district.

## Population, Materials and Methods

### Ethical Consideration and Recruitment

The study protocol was approved by the Ethics Committee of Kerman University of Medical Sciences and Health Services (protocol contract no.89/147 and ethic no.16/9/k).

The approved protocol included the screening procedures, interview and physical exam of the volunteers were performed by the personnel and the physicians of the research team.

Before the screening and selection of volunteers, several meetings with the household members and community leaders were organized and the aims, procedure of the study, potential benefits and harms were described. Candidates who were willing to participate and sign an informed consent were enrolled. Participants who contracted CL during the follow-up period were clinically examined and treated with meglumine antimoniate (Glucantime) alone or in combination with cryotherapy free of charge. In addition, individuals suspected of having any other disease were referred to the district hospital for further follow-up examinations and proper treatment.

### Study Design

This study was designed as a comparative cohort of two parallel groups of opium addicted and non-addicted resident of Bam district.

### Selection of Participants

**Figure 1:**
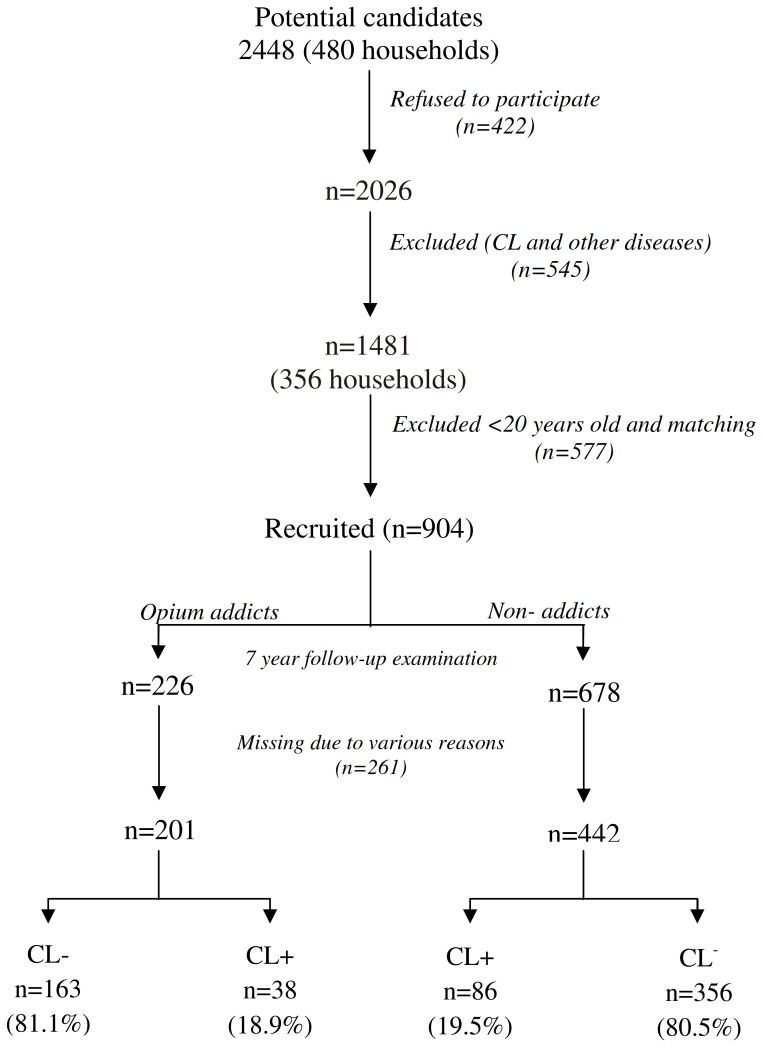
Study design.

The study design is summarized in Fig. 1. Initially, 2,448 household members were screened. A total of 480 households with 2,026 individuals, 1–78 years old were interviewed and physically examined by a team of experienced health workers for possible illness and history of opium consumption and active CL lesion or scars resembling CL. Approximately 100 households with 422 individuals refused to participate in the study. Altogether, 545 individuals with a history of CL or other diseases were excluded and 1,481 (73.1%) were eligible and included in the study and 577 (39%) were excluded due to the following reasons; age less than 20 years, age/sex matching and consent withdrawal or addiction after the earthquake. Overall, 904 eligible participants were included in the two arms of the study: opium addicted (n = 226) and non-addicted (n = 678). Finally, the participants were clinically examined and a questionnaire was completed for each individual, including demographic and clinical information.

### Definition of Opium Addicted Case

In this study, opium addiction was defined as an individual who consumes opium by oral inhalation on a daily basis for a period of at least 12 months. The addiction was identified by self-introduced through house-to-house visits and was clinically confirmed by an experienced physician based on the method proposed by Gilrame [20].

### Confirmation of CL

Slit-skin smear was taken from the edge of the clinically active CL lesion. Two glass slides were prepared; one for direct smear preparation, fixed with methanol and stained by Giemsa and the other smear was used for DNA extraction and the subsequent molecular identification of the species of the causative agent.

### Follow- up Examination

A clinic was specifically assigned and used for the management of the patients, including opium addicted and CL patients. This clinic was used as a headquarters for team activities such as meetings, reviewing of the study activities and also for implementation of preventive measures and research purposes. Active case-finding was done by households visit and clinical examination every 6 months for 7 years. Participants with suspected lesions which lasted for more than 2 weeks were referred for parasitological examinations as previously mentioned. The characteristics of the lesions such as number and size of the lesions, locations, onset and the severity of lesions were recorded. The size of indurations was measured using Sokal’s method [21].

### Molecular Identification

#### DNA preparation

DNA was collected from direct smears. Smear scrapings were transferred into micro tubes and washed (3000 rpm at 4°C for 10 min) three times using sterile PBS (pH = 7.2). DNA was extracted by proteinase k using the High Pure Template Purification Kit (Roche, Germany) according to the manufacture’s manual.

#### Nested – PCR

The nested-PCR was carried out as described by Noyes *et al*. 1998[22], using external primers CSB1XR (ATTTTTCGCGATTTTCGCAGAACG(and CSB2XF (CGAGTAGCAGAAACTCCCGTTCA) and specific internal primers 13Z (ACTGGGGGTTGGTGTAAAATAG) and LiR (TCGCAGAACGCCCCT) for amplification of variable minicircle fragments of *Leishmania* kDNA.

The PCR products were visualized by 1.5% agarose gel electrophoresis (Uvitech, Cambridge UK), using a 100- bp DNA size marker. *L. tropica* (MHOM/Sudan/58/OD strain), and *L. major* (MHOM/IR/02/Mash2 strain) were used as positive and double-distilled water as negative controls. *L. tropica* and *L. major* provided fragments of 750 bp and 560 bp, respectively. Confirmed cases were treated free of charge, according to National Guidelines.

### Statistical Analysis

Data was entered into a computer and standard statistical tests to determine the significance of differences between proportions (*χ* ^2^) and between means (t test) were used. The rate of CL incidence in the opium addicted and non-addicted cohorts were calculated as relative risk (RR), where RR is the ratio of the incidence rate in opium addicted group to that in non-addicted group. *P*<0.05 was considered as significance. The Kaplan-Meier survival curve and long-rank test, in Stata 11.2, were used to test equality of survival distribution between opium addicted and non-addicted groups.

## Results

Overall, 2,026 participants with mean age of 37.5±17.6 years, consisting of 1,025 males (50.6%) and 1,001 females (49.4%) were interviewed for a history of opium consumption, active lesions or scar of CL and health conditions (Table 1). Most of the opium addicted individuals were older than 21 years (mean age; 49.1±16.3 years) and opium addiction was significantly higher (*P*<0.001) in males than females and 249 participants (mean age 22.5±11.7 years) showed active CL lesions or scars which was more frequent in the age range 21–65 years than the other age groups. The rate of CL was higher in females than males. In addition, 9 participants showed history of acute or chronic diseases.

**Table 1:** Baseline characteristics of study participants.

Characteristic	PopulationNumber (%)	Opium addictionNumber (%)	CutaneousleishmaniasisNumber (%)
**Age (year) <6**	118(5.8)	0(0)	39(15.7)
** 6–20**	280(13.8)	6(2.1)	75(30.1)
** 21–65**	1379(68.1)	185(13.4)	117(47.0)
** >65**	249(12.3)	57(22.9)	18(7.2)
** Total**	2026(100.0)	248(100.0)	249(100.0)
**Sex Male**	1025(50.6)	231(93.1)	108(43.4)
** Female**	1001(49.4)	17(6.9)	141(56.6)

A total of 904 eligible individuals (mean age 44.5±9.1 years), including 226 opium addicted and 678 non–addicted volunteers were followed–up for development of CL lesion for a period of seven years from 2004 to 2010. Altogether, the drop-out rate during the follow up due to death and population displacement within the district, migration and habitual change in opium abuse was 29%. The two cohorts were similar in respect to age, sex and place of residency (Table 2). Most of the individuals were in the age range of 28–40 years (35.9%) and the least number was in age group of >52 years (29.1%) in the opium addicted and non – addicted groups. In the opium addicted group there were more males (93.1%) than females (6.9%).

**Table 2:** Age and sex distributions of opium addicted and non – addicted cohorts.

Characteristic	Examinedpopulation	Addicted*	Non – addicted*
**Age (year )**	Number (%)	Number (%)	Number (%)
** 28–40**	231(35.9)	70(34.8)	161(36.4)
** 41–52**	225(35.0)	72(35.8)	153(34.6)
** >52**	187(29.1)	59(29.3)	128(29.0)
**Sex**			
** Male**	618(96.1)	193(96.0)	425(96.2)
** Female**	25(3.9)	8(4.0)	17(3.8)

*There was no significant difference among the opium addicted and non-addicted with respect to age or gender.

The overall mean annual incidence rate of CL was 3% which was similarly distributed among the opium addicted and non-addicted individuals. A similar pattern of incidence was observed between the two cohorts (RR = 18.9/19.5 = 0.97) during the seven years follow up (Table 3). The lowest incidence rate was recorded in 2004, however it sharply increased thereafter and reached its highest level in 2006, which was coincident with the peak epidemic in Bam. On the other hand, during 2007–2010, the CL rate decreased steadily in both groups and there was no significant difference between the two groups.

**Table 3:** The incidence of cutaneous leishmaniasis cases in opium addicted and non – addicted cohorts by the year of contraction.

Year	Opium addicted*At risk population	Incidence No (%)	Opium non –addicted*At risk population	Incidence No (%)
**2004**	201	1(0.5)	442	3(0.7)
**2005**	200	3(1.5)	439	7(1.6)
**2006**	197	11(5.6)	432	22(5.1)
**2007**	186	9(4.8)	410	19(4.6)
**2008**	177	7(3.9)	391	14(3.6)
**2009**	170	4(2.3)	377	12(3.2)
**2010**	166	3(1.8)	365	9(2.5)
**Total**	1297	38(2.9)	2860	86(3.0)

*There was no significant difference between the incidence rates among the two cohorts.

**Figure 2:**
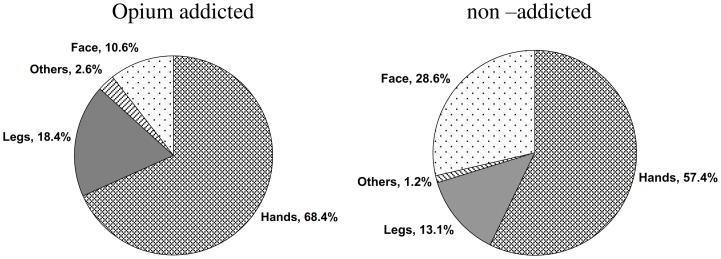
Location of the cutaneous leishmaniasis lesions in opium addicted and opium non – addicted cohorts.

**Figure 3:**
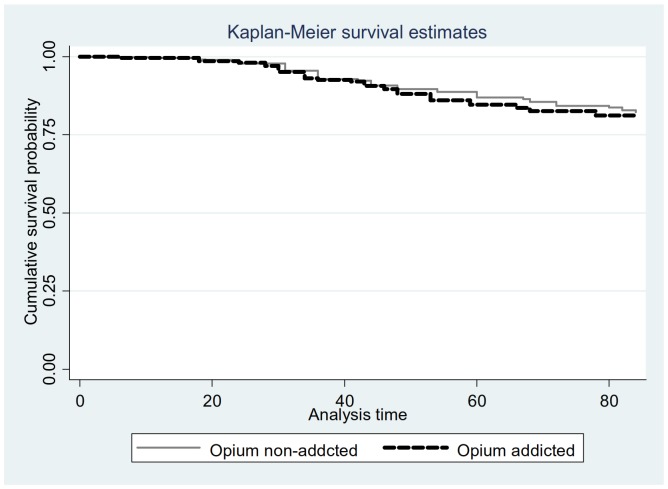
Kaplan-Meier survival estimates of cutaneous leishmaniasis in opium and opium non-addicted.

**Figure 4:**
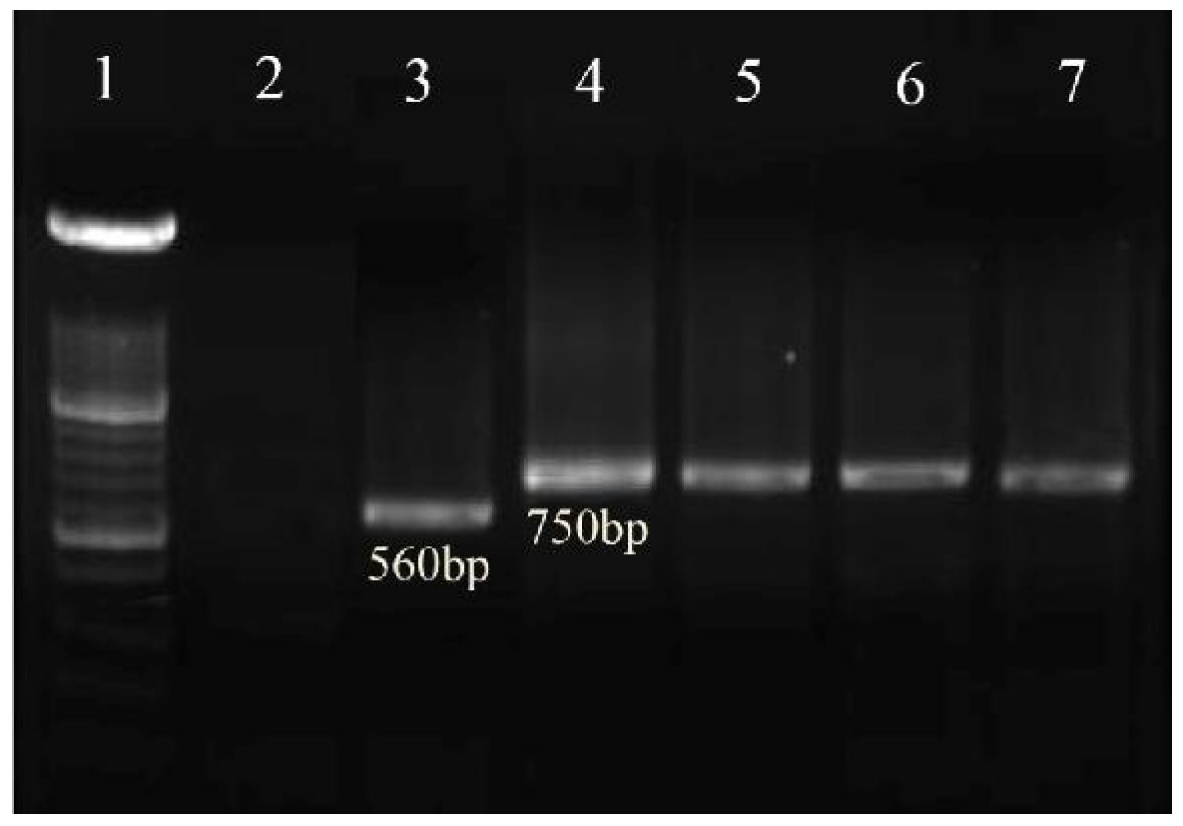
Agarose gel electrophoresis prepared for amplification of variable minicircle fragments of *Leishmania* kDNA by the nested PCR. Lane 1, DNA size marker, 100 bp; lane 2, negative control (distilled water); lane 3, *L.major* (positive control, 560 bp); lane 4, *L.tropica* (positive control,750 bp); lanes 5–7, *L.tropica* isolates obtained from the cutaneous leishmaniasis cases among opium addicted and opium non-addicted cohorts.

The severity of CL lesions was significantly different among the opium addicted and non–addicted patients (*P*<0.001, Table 4). The mean number of lesions (108 vs 104), the mean duration of the lesions (5 vs 3.6 months) and the mean diameter of the lesions (3.1 vs 2.1 cm) which were significantly higher in the opium addicted individuals compared to non–addicted CL patients. Similarly, the anatomical distribution of the lesions were significantly different (*P*<0.05) among the two groups (Fig. 2); in the opium addicted group the lesions were on the hands (68.4%), legs (18.4%), face (10.6%) and other locations (2.6%). In contrast, in non-addicted patients the lesion distribution was hands (57.4%), face (28.6%), legs (13.1%) and other sites (1.2%). There was no statistical significant difference (*P* = 0.7) in survival rates of opium addicted and non-addicted groups (Fig. 3). The results of nested-PCR showed 750bp fragments which corresponds with the *L. tropica* species (Fig. 4).

**Table 4:** The Severity of cutaneous leishmaniasis cases in opium addicted and non –addicted cohorts in terms of the number, duration and size of the lesions.

Characteristics		Opium addictedNo (% )	Opium non –addictedNo (% )
**No of lesions** *	1	22(57.9)	63(73.3)
	2	8(21.0)	13(15.1)
	≥3	8(21.1)	10(11.6)
**Duration of lesions** * **(month)**	<3	13(34.2)	45(52.3)
	3–6	11(28.9)	24(27.9)
	7–9	9(23.7)	13(15.1)
	>9	5(13.2)	4(4.7)
**Size of lesions** * **(cm)**	<1	10(26.3)	33(38.4)
	1–3	12(31.6)	39(45.3)
	4–5	9(23.7)	11(12.8)
	>5	7(18.4)	3(3.5)

*There was a significant difference between opium addicted and opium non –addicted cohorts (P<0.001).

## Discussion

Leishmaniasis remains a significant public health challenge in the Eastern Mediterranean Region and is endemic in 18 of the 23 countries of EMRO region including Iran [23]. Cutaneous leishmaniasis due to *L. tropica* has been reported in Bam since ancient times [9]. After the major earthquake of 2003, opium consumption has sharply increased in Bam, primarily as a result of psychological distress and post-traumatic stress disorder. The latter is a severe anxiety, developed after exposure to a major event such as an earthquake [24]. It was previously shown that disasters have significant psychological and psychiatric consequences [25].

Currently, the willingness toward substance misuse is a worldwide growing concern. The United Nations Office for Drug Control considers drug addiction as one of the quadruplet crises in the world and categorized Iran among the high-risk countries [3]. The main reason for increasing frequency of opium misuse appears to be in large part due to increased demands for treatment of chronic or acute pain [16]. The reasons for a seven-year follow-up strategy was due to a high proportion of the population having a CL scar. A mean scar rate of 14% was observed for the district, while it was 40% in hyperendemic areas. Moreover, the scarcity of the volunteer opium addicted participants was another limiting factor.

The incidence rate of CL was similar in the two groups which indicate that they share similar risk factors for CL. In fact, there was no significant difference in CL incidence between the opium addicted and non-addicted groups during follow up. The selected populations were mainly indigenous and fairly stable, with similar exposure rate to sand fly bites. The reason that the incidence rate of CL increased significantly in 2006 is due to a variety of environmental changes occurring after the earthquake which affect the risk factors. Modification of the physical environment following a major earthquake had a profound effect on the abundance of vectors, level of transmission and creation of fly breeding sites [11], [13].

Severity of CL lesions in terms of the number, duration and size of the lesion was significantly higher in opium addicted patients as compared to non-addicted patients. The reason for the difference in severity of the lesion is not known. However, there is evidence indicating that alkaloid opoids such as morphine and heroin induce a significant immunosuppressive effect [26], [27] and morphine depresses phagocytic cell function [28], natural killer cell activity [29], delayed- type hypersensitivity [30], antibody formation [31] and reactive oxygen and nitrogen intermediates by phagocytes [28], [32]. Morphine is known to be an immunomodulator in a mouse model of *Leishmania donovani* [33] and *Toxoplasma gondii* infections [34]. In addition loss of appetite, as a main risk factor, obviously restricts the availability of nutrients to drug abusers [35]. Such a deprivation of food intake, in turn, could have a deleterious effect on the addicts immune system. Other precipitating factors include low motivation for treatment, perceived stigma or fear of narcotic withdrawal if hospitalized all of which may also play a role in the severity of the lesions.

Location of the lesions was different before and after the earthquake. Lesions were more frequent on the face before earthquake and more frequent on the hands after the earthquake in the both groups. The lesions were more frequent on hands and legs in opium addicts compared to non-addicted individuals and the majority of the lesions were on hands and face in non-addicted patients. The reason is not known but might be related to the behaviour of the drug abusers, adult sand flies are mainly nocturnal and the peak activity is commonly after sunset which is the time that most addicts smoke opium [36].

In conclusion, the current findings strongly suggest that in endemic foci, major disasters such as earthquakes might create a variety of risk factors, which in turn provide a suitable condition for transmission of CL and intensification of opium consumption. The chance of contracting ACL due to *L. tropica* was similar among opium addicted and non-addicted individuals however, the lesions were significantly more severe in opium addicts than in non-addicted patients.
